# CaBind_MCNN: Identifying Potential Calcium Channel Blocker Targets by Predicting Calcium-Binding Sites in Ion Channels and Ion Transporters Using Protein Language Models and Multiscale Feature Extraction

**DOI:** 10.1021/acs.jcim.4c02252

**Published:** 2025-02-07

**Authors:** Yan-Yun Chang, Yu-Chen Liu, Wei-En Jhang, Sin-Siang Wei, Yu-Yen Ou

**Affiliations:** † Department of Computer Science and Engineering, Yuan Ze University, Chung-Li 32003, Taiwan; ‡ Graduate Program in Biomedical Informatics, Yuan Ze University, Chung-Li 32003, Taiwan

## Abstract

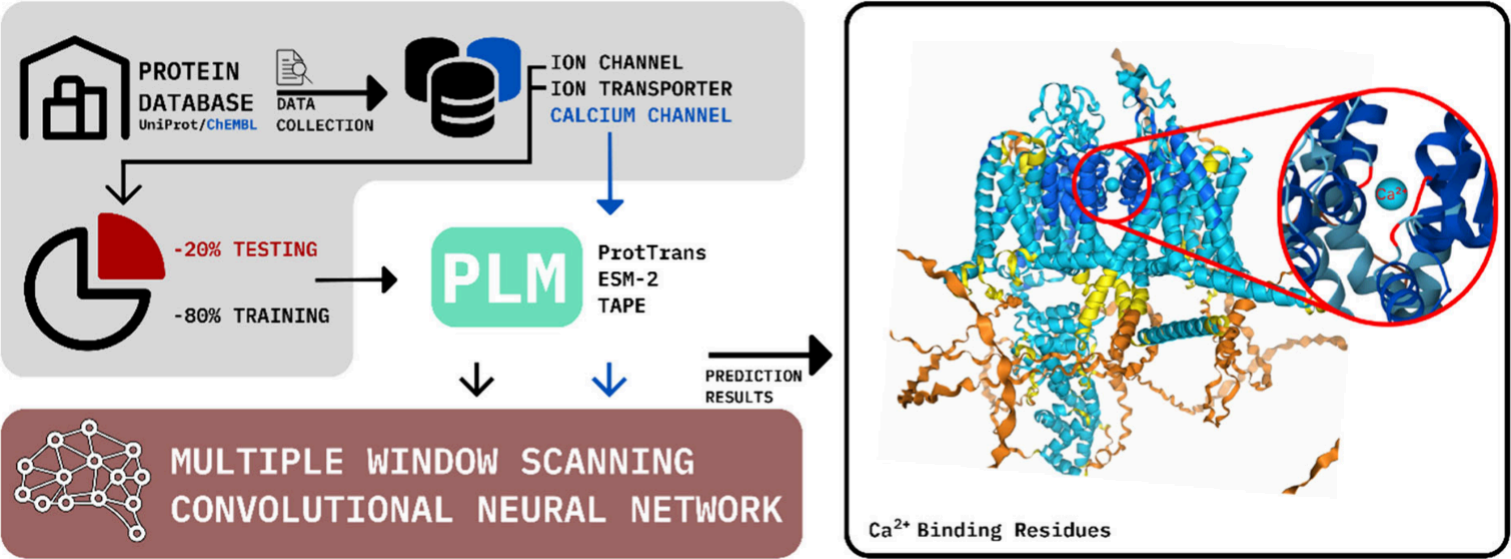

Calcium ions (Ca^2+^) are crucial for various physiological processes, including neurotransmission and cardiac function. Dysregulation of Ca^2+^ homeostasis can lead to serious health conditions such as cardiac arrhythmias and hypertension. Ion channels and transporters play a vital role in maintaining cellular Ca^2+^ balance by facilitating Ca^2+^ transport across cell membranes. Accurate prediction of Ca^2+^ binding sites within these proteins is essential for understanding their function and identifying potential therapeutic targets, particularly for developing novel calcium channel blockers (CCBs). This study introduces CaBind_MCNN, an innovative computational model that leverages pretrained protein language models (PLMs) and a multiscale feature extraction approach to predict Ca^2+^ binding sites in ion channels and transporter proteins. Our method integrates embeddings from the ProtTrans PLM with a convolutional neural network (CNN)-based multiwindow scanning approach, capable of capturing diverse sequence features relevant to Ca^2+^ binding. The model, trained on a curated data set of 27 calcium-binding protein sequences, achieves high accuracy with an area under the curve (AUC) of 0.9886, significantly outperforming some existing methods. These results demonstrate the potential of CaBind_MCNN to enhance drug discovery efforts by identifying potential CCB targets and advancing the development of novel therapies for calcium-related disorders.

## Introduction

Calcium (Ca^2+^) is the most abundant mineral in the human body, with 99% stored in bones and teeth, while the remaining 1% is distributed in blood, muscle, nerve cells, and other tissues. Calcium plays a crucial role in maintaining overall health by regulating various physiological processes through its interaction with different proteins. These processes include neurotransmission,^1,2^ muscle contraction, and relaxation, particularly in the heart. Calcium’s role in cardiac functions^3−5^ is mediated through ion channels and transporter proteins, which are integral components of the cell membrane responsible for the transmembrane movement of calcium ions.

**Figure 1:**
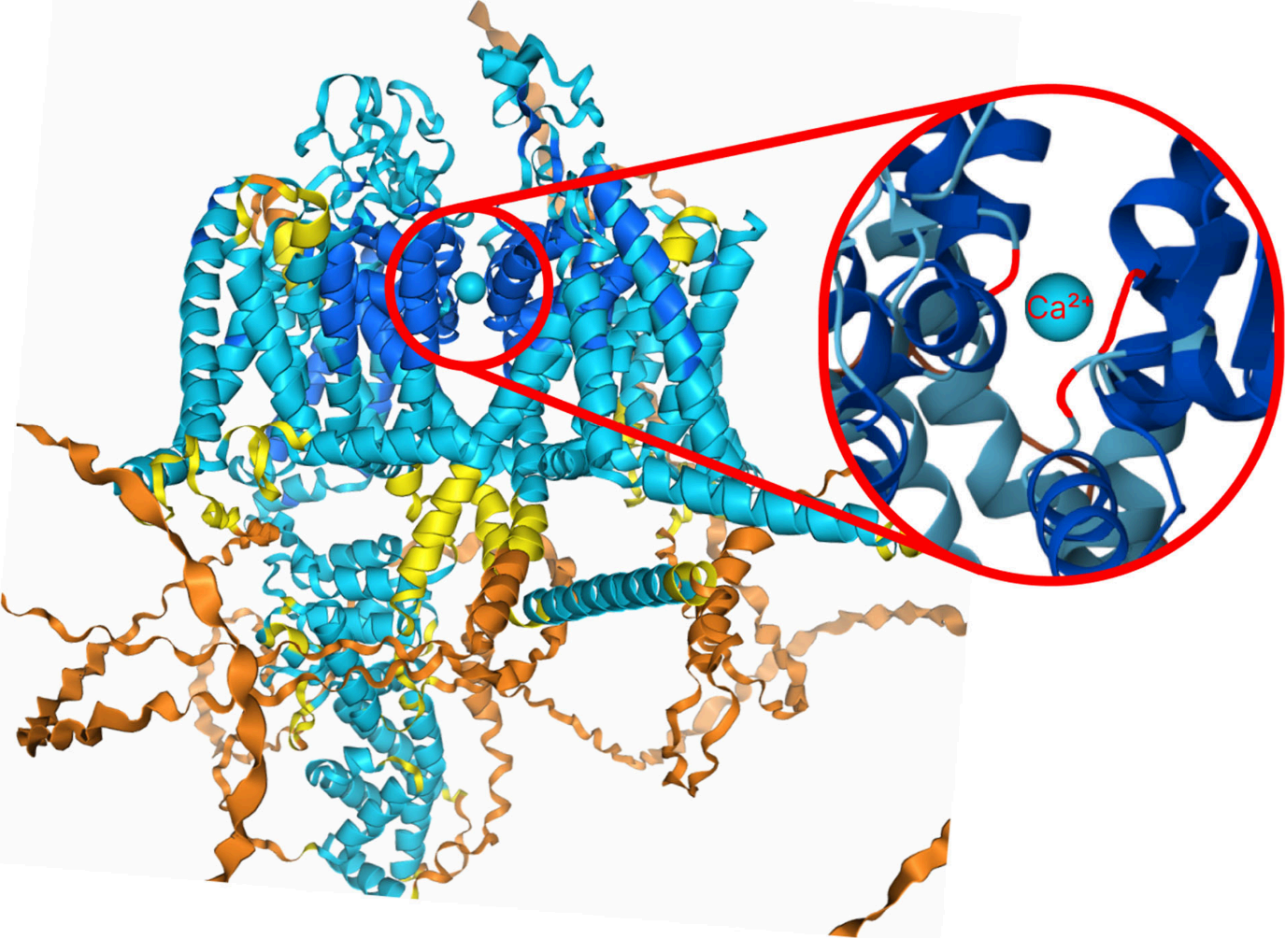
Red areas show where the channel binds to calcium.

Ion channels^6,7^ and ion transporter proteins^8^ are essential for cellular function, controlling the movement of molecules and ions across cellular membranes. Their fundamental roles in cellular physiology make them key drug targets. Dysfunction of these proteins is directly associated with a wide range of diseases, including cardiovascular, neurological, and metabolic disorders, as well as cancer. Computational advancements^9,10^ have significantly improved our ability to accurately identify these crucial membrane proteins. Ion transporter proteins, such as the sodium–calcium exchanger (NCX)^11,12^ in cardiac myocytes, maintain essential intracellular environments by regulating calcium efflux. Similarly, ion channels, like calcium channels (Figure 1), provide selective pathways for ion flow, with intricate binding sites controlling permeation and electrochemical gradients vital for signaling.^13−15^ The strong link between malfunctioning ion channels and transporters and various diseases makes them attractive targets for therapeutic development.

Arrhythmia^16−18^ is one of the most common heart conditions, characterized by irregular heart rhythms due to abnormal cardiac signal conduction. The mechanisms behind this condition are extremely complex and difficult to predict. It is closely related to other cardiovascular diseases, potentially leading to heart failure and sudden death. Excessive accumulation of calcium ions is considered a key factor in inducing apoptosis in cardiomyocytes apoptosis,^18,19^ resulting in arrhythmias. Clinically, to address these heart issues caused by calcium ion imbalances, a class of drugs known as calcium channel blockers (CCBs) has been developed.

**Figure 2:**
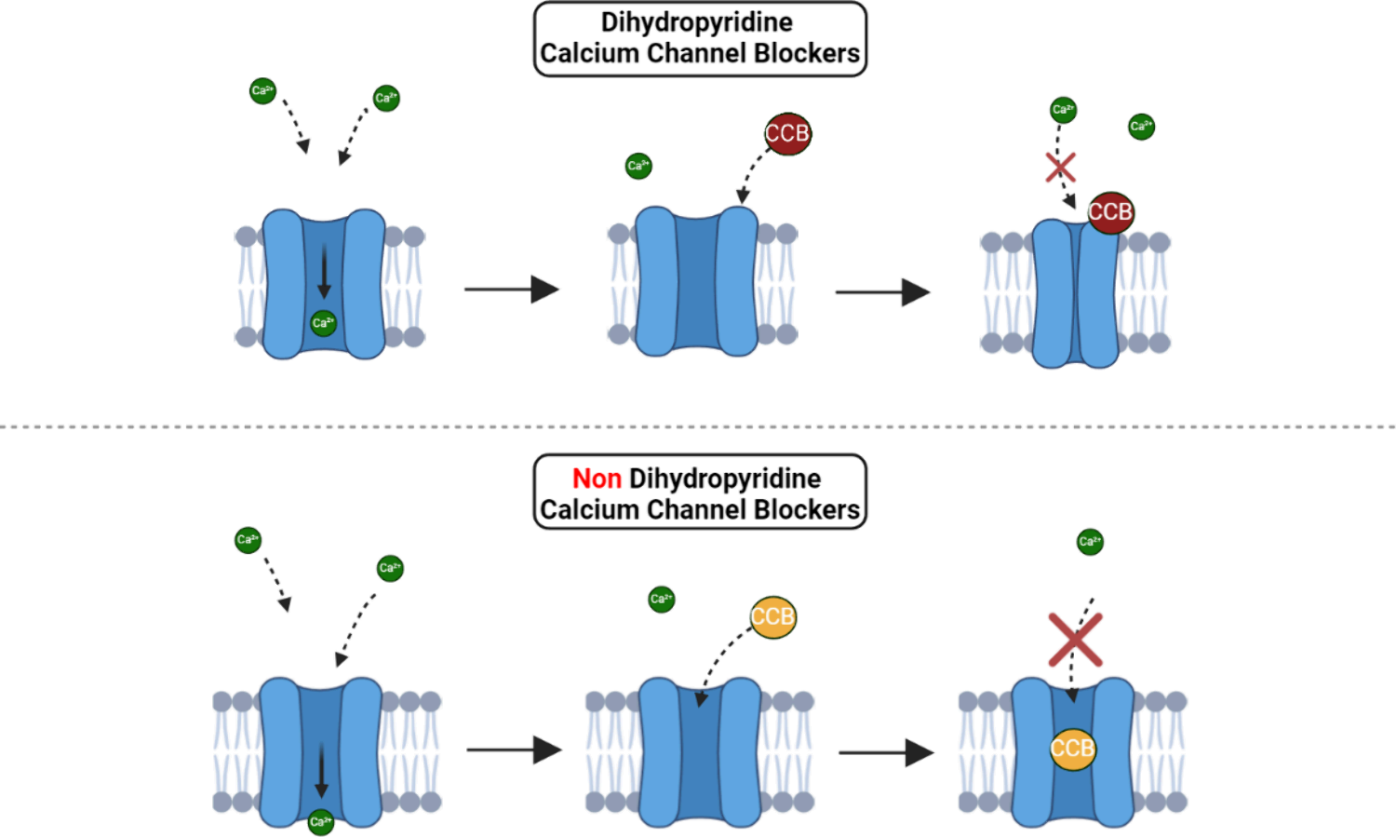
Two types of CCBs and their mechanisms of action

In Figure 2, CCBs^20,21^ work by binding to calcium channel proteins and altering their structure to prevent calcium ions from entering the cell, decreasing intracellular calcium concentrations. This results in the relaxation of vascular smooth muscle, reduction of blood pressure, and alleviation of symptoms such as palpitations and arrhythmias. However, there are still some limitations to the clinical application of current CCBs.^22,23^ Some patients may have poor tolerance to these drugs, potentially experiencing side effects such as hypotension, dizziness, and fatigue that limit the long-term use of CCBs. Additionally, some CCBs may reduce the heart’s contractility, leading to incomplete cardiac output, which is particularly detrimental for patients with heart failure. Furthermore, when CCBs are used in combination with other medications, they may excessively lower the heart rate or result in an atrioventricular conduction block.

**Figure 3:**
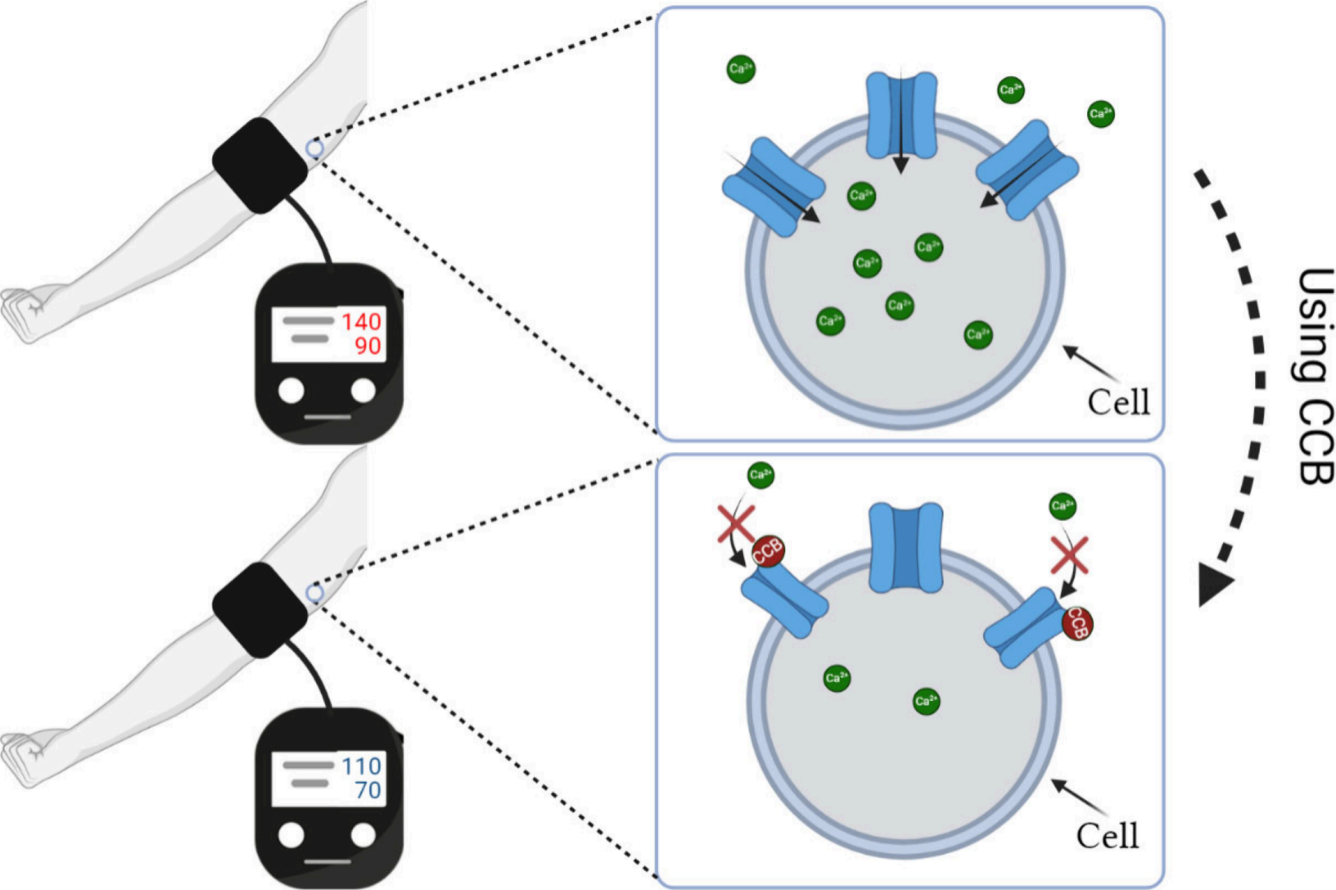
Effect of CCBs on hypertension.

While existing CCBs have played a crucial role in treating (such as arrhythmias, hypertension as shown in Figure 3, and angina) or preventing (such as stone heart) various heart-related diseases, their limitations are numerous, and they may not be safe for all patients. In this context, our CaBind_MCNN model can provide significant assistance in discovering new CCBs by predicting their calcium binding sites. This model can accurately predict the binding positions of calcium ions on different channel proteins and transporter proteins, which not only helps identify novel potential drug targets but may also reveal further details about existing drugs’ mechanisms. For example, by analyzing these binding sites, researchers can design drugs specifically targeting certain calcium channel varieties, or adjust the structure of existing CCBs to improve their specificity and effectiveness, thus reducing side effects.

Several other research groups have also proposed methods for predicting Ca^2+^ ion binding positions. For instance, Ying Xia’s team developed GraphBind,^24^ which utilizes graph neural networks (GNNs) to construct graphs based on a protein’s structural context. Their approach involves building graphs of the highly neighboring residues around a target residue, followed by applying hierarchical graph neural networks (HGNNs) to embed structural and latent features for binding residue recognition. Another example is Jian Zhang’s team’s method, SCAMPER,^25^ which leverages the BioLip database, enhancing data quality through re-editing. SCAMPER’s architecture consists of two layers: an initial layer for basic predictions and a second layer to rank the results from the first layer, employing 5-fold cross-validation to minimize prediction errors. While these models offer valuable approaches, they can be computationally intensive and time-consuming. To address this, we propose CaBind_MCNN, a model designed for high prediction accuracy and efficient computation, by combining current popular pretrained language models with multiwindow scanning.

CaBind_MCNN, a novel model that leverages the power of pretrained protein language models (PLMs) and multiwindow scanning to achieve high prediction accuracy with reduced computational complexity. This approach can facilitate the identification of potential CCB targets by pinpointing Ca^2+^ binding sites in ion channels and transporter proteins, paving the way for developing of novel therapies. In this study, we demonstrate the effectiveness of CaBind_MCNN in accurately predicting Ca^2+^ binding sites within VGCCs and NCXs. We show that our model outperforms existing methods, particularly in sensitivity, highlighting its potential for discovering novel CCB binding sites and advancing drug development for calcium-related disorders. Our findings provide valuable insights into the molecular mechanisms of Ca^2+^ regulation and offer a powerful tool for accelerating the discovery of new therapeutic targets.

## Materials and Methods

**Figure 4:**
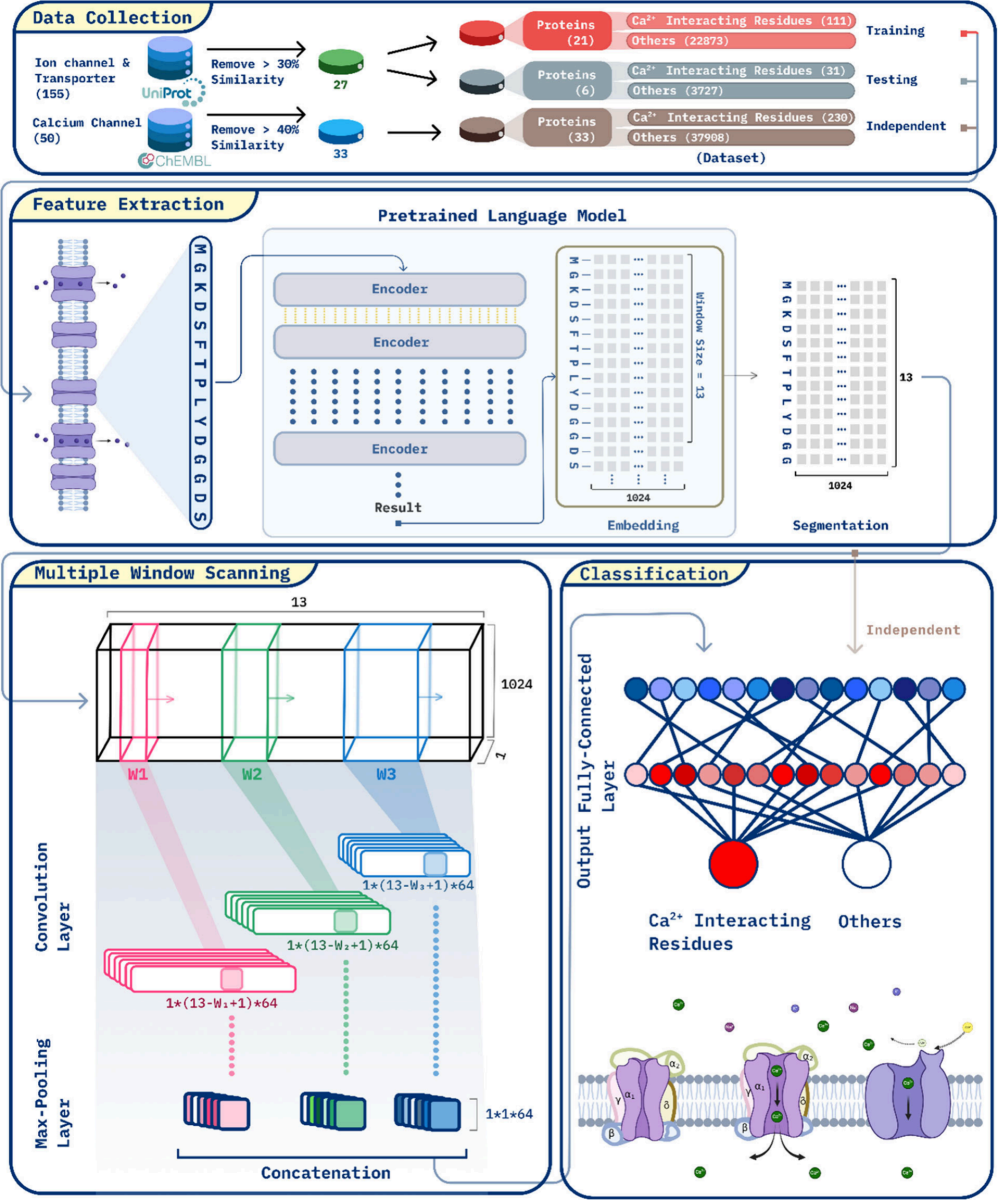
Workflow for Ca^2+^ binding site prediction model.

In Figure 4, this figure presents the workflow for predicting calcium binding sites in ion channels and transporter proteins. The process begins with data collection from the UniProt database, followed by preprocessing to ensure a diverse data set for training and validation. Protein sequences are then transformed into high-dimensional embeddings using a pretrained protein language model (such as ProtTrans), capturing key biological signals. The multiple window scanning neural network (MCNN) scans these embeddings at various scales, identifying potential calcium-binding residues. The extracted features are classified to distinguish between calcium-interacting and noninteracting residues, with predictions validated against independent data sets to ensure robustness and accuracy. This workflow showcases the effective integration of protein language models and deep learning to improve protein function prediction.

## Data Collection

Our study focused on identifying calcium-binding sites in proteins, with a particular emphasis on ion channels and transporters relevant to calcium channel blocker (CCB) activity. Data was sourced from multiple databases and curated to ensure high quality and minimize redundancy.

### Initial Data Set: Ion Channels and Transporters

Initially, we focused on ion channel and transporter proteins known to bind calcium ions, sourcing our data from the UniProt database. We identified 155 relevant protein sequences. To maintain diversity and reduce redundancy, we used the CD-HIT tool for similarity comparison, retaining only those sequences with less than 30% sequence identity. This process yielded an initial data set of 27 sequences, containing 142 calcium-binding residues and 26,600 nonbinding residues. This data set was partitioned into training and validation subsets using an 80:20 ratio. The training set comprised 21 sequences (111 calcium-binding residues, 22,873 nonbinding residues), and the validation set contained 6 sequences (31 calcium-binding residues, 3,727 nonbinding residues).

### Independent Test Set: Calcium Channel Blocker Targets from ChEMBL

To further enhance the robustness and pharmacological relevance of our study, and following suggestions to validate our methods against well-characterized drug targets, we constructed an additional independent test set derived from the ChEMBL database. We searched ChEMBL for proteins annotated as targets for Calcium Channel Blockers (CCBs). This search yielded 50 UniProt entries. To ensure data set diversity and reduce redundancy, we applied a 40% CD-HIT similarity threshold, resulting in a final data set of 23 calcium channel protein sequences. This data set provides a valuable resource for evaluating our model’s ability to predict calcium-binding sites in proteins directly relevant to CCB activity and allows for a more comprehensive comparison with existing prediction methods. The detailed breakdown of this data set is provided in Table 1.

**Table 1:** Statistics of the Survey Data Set

data set	protein sequence with Ca^2+^ binding residue	similarity < 30%	training	testing
ion channel/ion transport	155	27	21	6

data set	protein sequences	Ca^2+^ binding residues	nonbinding residues
training data	21	111	22873
testing data	6	31	3727

data set	protein sequence with Ca^2+^ binding residue	similarity < 40	Ca^2+^ binding residues	nonbinding residues
calcium channel from ChEMBL	50	23	177	23640

Table 1 summarizes the composition of all data sets used in this study. While the initial data set of ion channels and transporters is relatively small (27 sequences after redundancy removal), this is due to the stringent requirement for experimentally validated calcium-binding sites within these specific protein classes. The addition of the ChEMBL-derived data set significantly expands our evaluation by focusing on proteins directly involved in CCB interactions, thus enhancing the translational relevance of our findings and addressing concerns about the initial data set’s size. All data sets underwent rigorous curation to ensure high quality and minimize redundancy.

### Embedding Generation with Pretrained Protein Language Models

To capture the complex sequence patterns and structural information crucial for predicting calcium-binding sites, we used protein PLMs from the ProtTrans^26^ series. PLMs are powerful tools based on the pretraining concept from natural language processing, applied to protein sequence analysis. These models undergo unsupervised pretraining on large-scale protein sequence data sets, acquiring structural and functional information within protein sequences. This allows them to generate high-dimensional embeddings that capture the subtle differences and contextual dependencies of the sequences.

ProtT5-XL-UniRef50, an advanced protein language model based on the Transformer architecture, was pretrained on a deredundant protein sequence collection, ProtT5-XL-UniRef50, enabling the ProtTrans model to learn the common features prevalent across diverse protein families. The high-dimensional embedding vectors generated by the ProtT5-XL-UniRef50 model (up to 1024 dimensions) encapsulate rich sequence features, which include not only basic amino acid sequence information but also deeper structural and functional insights. This enables ProtTrans to make more detailed and accurate predictions than traditional methods based on manual feature identification, particularly when dealing with complex biological problems. By identifying contextual information within sequences, ProtTrans can identify potential functional domains and interaction sites, which is essential for predicting key biological functions like calcium-binding sites.

### Multiscale Feature Extraction with Multiple Window Scanning

We implemented a multiple window scanning technique,^27^ an extension of convolutional neural networks (CNNs), to extract features at varying scales across the protein sequences. This method involves scanning the sequence embeddings with windows of different sizes to capture both local and global sequence features. For each window size, the CNN computes the weighted sum over overlapping regions, generating a vector representation for each window. We applied max-pooling to each vector to retain the most significant features, which were then concatenated and passed through a fully connected layer to produce the final predictions.

The choice of window sizes was carefully optimized based on performance metrics, with window lengths ranging from 2 to 12. Our experiments revealed that a combination of windows sizes 6, 8, 10, and 12 yielded the highest predictive accuracy, balancing the capture of local and global features.

## Performance Evaluation

The predictive model was trained using the training data set, with hyperparameters tuned to maximize performance on the validation set. The model’s effectiveness was evaluated using standard metrics, including sensitivity, specificity, accuracy, matthews correlation coefficient (MCC), and area under the receiver operating characteristic curve (AUC). Sensitivity and specificity were particularly critical for assessing the model’s ability to correctly identify calcium-binding and nonbinding residues, respectively.

To validate the robustness of our model, we conducted additional experiments comparing its performance with different filter sizes, window lengths, and feature sets, including protein PLMs, ESM-2, and TAPE embeddings. The results consistently demonstrated the superiority of our protein PLMs-based model, particularly in independent test scenarios, where it achieved an AUC of 0.9886, significantly outperforming other models.
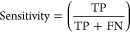
1
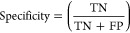
2
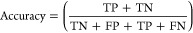
3
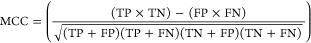
4

These metrics are based on the classification of predictions into four categories: True Positives (TP), True Negatives (TN), False Positives (FP), and False Negatives (FN). Sensitivity, also known as recall, measures the proportion of actual positive cases (Ca^2+^ binding residues) correctly identified by the model. Specificity quantifies the proportion of actual negative cases (nonbinding residues) correctly classified. Accuracy reflects the overall proportion of correct predictions, regardless of the class. MCC provides a balanced measure of the model’s performance, considering all four classification categories, and ranges from −1 (total disagreement between prediction and observation) to +1 (perfect prediction), with 0 indicating random performance. AUC, calculated as the area under the ROC curve, represents the model’s ability to distinguish between positive and negative cases across different classification thresholds. An AUC value of 1 signifies perfect discrimination, while a value of 0.5 indicates random classification. Our model’s performance in predicting calcium binding sites within ion channels and transporter proteins was rigorously evaluated using the above four metrics. Among these, AUC was our primary evaluation criterion, given its robustness in summarizing the model’s performance across different threshold settings.

### The Performance Comparison with Different Single Window Sizes

We first analyzed the impact of different single window sizes on model performance. As shown in Table 2, we observed a gradual increase in AUC values as the window size increased, with a peak of 0.9796 at a window size of 12. This trend suggests that larger windows more effectively capture sequence features relevant to calcium-binding sites, as they encompass a broader sequence context, thereby providing a more comprehensive reflection of calcium-binding site background information. However, once the window size reaches a certain size, performance gains diminish. This may be due to the introduction of irrelevant sequence information, which negatively impacts prediction accuracy. Therefore, adjusting the window size must consider the effectiveness and relevance of the sequence information to ensure that the model captures valid information while minimizing the influence of irrelevant data. Optimizing window size therefore becomes a critical step in improving model accuracy.

**Table 2:** Performance Comparison with Different Single Window Sizes

single window sizes	sensitivity	specificity^18,19^	accuracy	MCC	AUC
2	0.9034	0.9028	0.9028	0.1992	0.9479
4	0.9148	0.9062	0.9062	0.2046	0.9481
6	0.9241	0.9136	0.9137	0.2110	0.9607
8	0.9282	0.9261	0.9261	0.2281	0.9661
10	0.9436	0.9379	0.9379	0.2642	0.9748
12	0.9785	0.9284	0.9286	0.2527	0.9796

### The Performance Comparison with Different Window Combinations

Next, we further evaluated model performance using combinations of different window sizes. The window selection order was based on the AUC ranking from high to low in Table 2. As shown in Table 3, the combination of window sizes 6, 8, 10, and 12 achieved the highest AUC value of 0.9860. This result underscores the advantages of a multiscale approach, where different window sizes identify complementary aspects of sequence features, leading to more comprehensive and accurate predictions. The success of multiwindow combinations illustrates the complexity of calcium-binding sites, which may involve interactions across multiple sequence scales. While a single window can only capture sequence information at a specific scale, multiple windows can integrate information from different sizes. This allows the model to capture local motifs and recognize broader sequence patterns, greatly enhancing its predictive capabilities.

**Table 3:** Performance Comparison with Different Window Combinations^a^

window combinations	sensitivity	specificity	accuracy	MCC	AUC
[12]	0.9785	0.9284	0.9286	0.2527	0.9796
[12, 10]	0.9618	0.9443	0.9444	0.2994	0.9810
[12, 10, 8]	0.9743	0.9497	0.9498	0.3099	0.9849
**[12, 10, 8, 6]**	**0.9828**	**0.9546**	**0.9548**	**0.3206**	**0.9860**
[12,10, 8, 6, 4]	0.9597	0.9505	0.9506	0.2857	0.9824
[12,10, 8, 6, 4, 2]	0.9618	0.9541	0.9541	0.3085	0.9820

a The bolded row indicates the configuration selected for the final model.

### Optimizing the Number of Convolutional Filters

We investigated the impact of the number of convolutional filters on model performance. As shown in Table 4, we evaluated filter sizes ranging from 64 to 2048, using the optimal multiwindow configuration determined previously. A filter size of 64 achieved the highest AUC (0.9886), along with excellent sensitivity (0.9799) and specificity (0.9636). Increasing the number of filters beyond 64 did not yield further improvements and, in some cases, slightly reduced performance. This observation highlights the critical balance between model complexity and generalization ability in deep learning. While more filters can capture richer features, an excessively complex model risks overfitting to the training data, hindering its ability to generalize to unseen examples. Therefore, 64 filters provided the optimal balance, allowing the model to learn relevant patterns without overfitting.

**Table 4:** Performance Comparison with Different Filter Sizes^a^

filters sizes	sensitivity	specificity	accuracy	MCC	AUC
**64**	**0.9799**	**0.9636**	**0.9637**	**0.3479**	**0.9886**
128	0.9789	0.9656	0.9657	0.3480	0.9882
256	0.9789	0.9556	0.9557	0.3153	0.9875
512	0.9751	0.9582	0.9583	0.3216	0.9857
1024	0.9828	0.9546	0.9548	0.3206	0.9860
2048	0.9789	0.9486	0.9487	0.3082	0.9855

a The bolded row indicates the configuration selected for the final model.

This filter size optimization, combined with the multiwindow strategy, culminated in a robust and accurate calcium-binding site predictor. Starting with a single-window approach (Table 2, AUC 0.9796), we progressively improved performance through the multiwindow strategy (Table 3, AUC 0.9860) and finally achieved peak performance with the optimized filter size (Table 4, AUC 0.9886). These incremental gains, while seemingly small, collectively represent a significant advancement in predictive capability. The optimized filter size ensures the model learns generalizable patterns from the data rather than memorizing specific examples, enhancing the reliability of predictions on new sequences.

The true impact of these optimizations becomes clear when compared to existing state-of-the-art methods (Table 7). Our final model significantly outperforms these approaches, validating the effectiveness of our design choices and demonstrating the substantial contribution of each refinement step. This improved performance provides researchers with a powerful new tool for investigating calcium-binding proteins and their diverse biological functions, particularly in the context of drug discovery.

### The Performance Comparison with Different Window Length

According to the data in Table 5, the impact of different window lengths on model performance shows that the model achieved the best results with a window length of 6 and an AUC value of 0.9886. As the window length increased, the AUC generally decreased, suggesting that longer windows might introduce redundant information, thereby diluting predictive features. This finding suggests that calcium-binding site prediction should focus more on specific, localized sequence features. Overly broad contextual information may not only be unhelpful but possibly also interfere with the model’s ability to identify core features. Therefore, determining an appropriate window length that maximizes the capture of functionally relevant local sequence features while avoiding adding unnecessary information is crucial for improving the model’s predictive accuracy.

**Table 5:** Performance Comparison with Different Splitting Window Length^a^

window length	sensitivity	specificity	accuracy	MCC	AUC
**6**	**0.9799**	**0.9636**	**0.9637**	**0.3479**	**0.9886**
7	0.9846	0.9443	0.9445	0.2863	0.9842
8	0.9549	0.9471	0.9471	0.2798	0.9780
9	0.9674	0.9434	0.9435	0.2766	0.9796

a The bolded row indicates the configuration selected for the final model.

### The Performance Comparison with Different Features

In this study, we selected ProtT5 from the ProtTrans series, specifically ProtT5-XL-UniRef50, as the primary language model. This model was pretrained on the UniRef50 data set, a nonredundant protein sequence database provided by UniProt, where all sequences share less than 50% similarity. For ESM-2, we selected two models with different dimensions: the 1280-dimensional model (esm2_t33_650M_UR50D) and the 2560-dimensional model (esm2_t36_3B_UR50D). On the other hand, TAPE employed the Bert-based model with a 768 dimension.

As shown in Table 6, after 5-fold cross-validation and comparison, ProtT5-XL-UniRef50 achieved the highest AUC (0.9886), demonstrating the highest performance. It is worth noting that the higher-dimensional ESM-2 model exhibited overfitting tendencies in the calcium ion binding site prediction task, suggesting that larger models may not generalize well to our data set. In contrast, the TAPE model, due to its smaller dimension, achieved an AUC of only 0.9606, which was insufficient in terms of accuracy. Ultimately, after extensive comparisons, ProtTrans emerged as the best method for our study.

**Table 6:** Performance Comparison with Different Features^a^

feature sets	sensitivity	specificity	accuracy	MCC	AUC
ProtT5-XL-UniRef50	**0.9799**	**0.9636**	**0.9637**	**0.3479**	**0.9886**
ESM-2-650M	0.9584	0.9655	0.9655	0.3564	0.9857
ESM-2-3B	0.9077	0.9314	0.9313	0.2677	0.9490
TAPE	0.9319	0.9138	0.9139	0.2104	0.9606

a The bolded row indicates the configuration selected for the final model.

**Figure 5:**
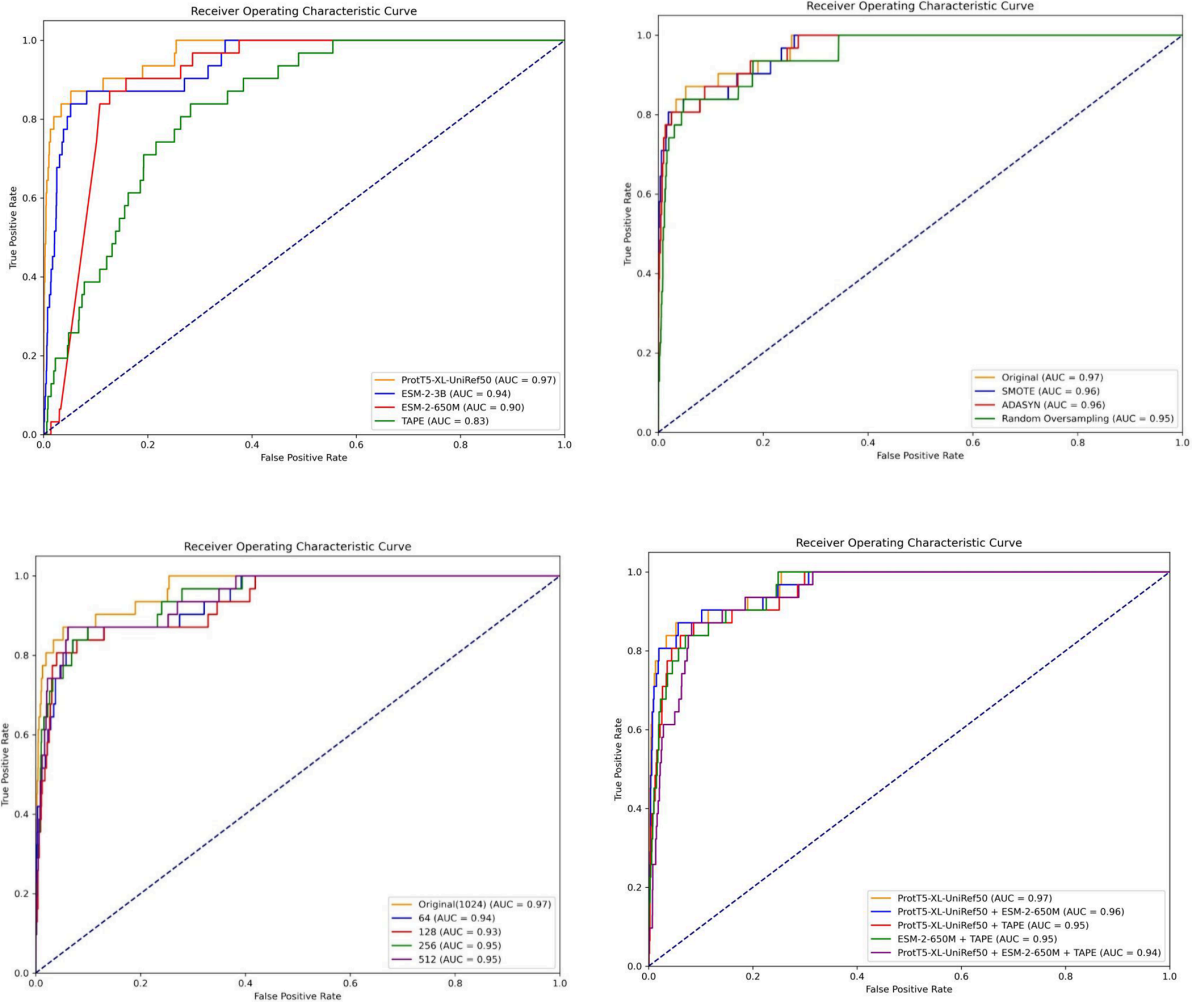
(a) ROC curve for performance with different features. (b) ROC curve for performance with different imbalance-handling techniques. (c) ROC curve for performance with different PCA dimensions. (d) ROC curve for performance with merged features.

Next, we further analyzed the model’s performance regarding TP, FP, TN, and FN in independent tests. As shown in Table 7 and Figure 5-a, ProtT5-XL-UniRef50 and TAPE had TP values of 26. ESM-2 (1280 and 2560 dimensions) achieved slightly higher TP values of 27. However, the difference in TP between the four models was minimal. Notably, the FP for ProtT5-XL-UniRef50 was only 80, which is 229 fewer than ESM-2-650M, 393 less than ESM-2-3B, and 971 less than TAPE.

**Table 7:** Performance Comparison with Different Features on the Testing Data Set

feature sets	TP	FP	TN	FN	sensitivity	specificity	accuracy	MCC	AUC
ProtT5-XL-UniRef50	26	80	3647	5	0.8387	0.9785	0.9774	0.4465	0.9718
ESM-2-650M	27	309	3418	4	0.8710	0.9824	0.9815	0.4982	0.9627
ESM-2-3B	27	473	3254	4	0.9032	0.9230	0.9228	0.3330	0.9622
TAPE	26	1051	2676	5	0.7742	0.8298	0.8293	0.1467	0.8768

Although TP reflects the model’s ability to correctly identify binding sites, increasing TP often leads to an increase in FP. This can be indirectly verified through changes in sensitivity and specificity. When sensitivity increases, specificity decreases. An increase in FP implies higher resource demands during drug development.

### The Performance Comparison with Different Imbalance-Handling Techniques on the Testing Data Set

Considering the significant imbalance between positive and negative samples in the initial data set, capturing true positives and balancing the reduction of false positives becomes particularly crucial in such data sets. We applied additional imbalance treatments to the data set and compared the results with the original method. The imbalance treatment methods included SMOTE,^28^ ADASYN,^29^ and Random Oversampling.

As shown in Table 8 and Figure 5-b, imbalance treatments improved sensitivity to some extent (e.g., ADASYN increased sensitivity from 0.8387 to 0.8710 in the test set). However, this was often accompanied by a significant increase in FP, as well as a decrease in specificity and MCC. For example, ADASYN resulted in an FP of 330, a drop in specificity to 0.9115, and a decrease in MCC to 0.2414. This “increase in TP at the expense of specificity” is particularly detrimental to drug development, as each false positive could become a false target during subsequent experimental stages, leading to unnecessary resource investment and wasted manpower. To achieve a balance between TP and specificity while maintaining model stability and preventing overfitting, we selected the original method as the final version.

**Table 8:** Performance Comparison with Different Imbalance-Handling Techniques on the Testing Data Set

imbalance-handling	TP	FP	TN	FN	sensitivity	specificity	accuracy	MCC	AUC
original	26	80	3647	5	0.8387	0.9785	0.9774	0.4465	0.9718
SMOTE	26	178	3549	5	0.8387	0.9522	0.9513	0.3157	0.9638
ADASYN	27	330	3397	4	0.8710	0.9115	0.9111	0.2414	0.9638
random oversampling	26	182	3545	5	0.8387	0.9512	0.9502	0.3124	0.9514

The original method maintained a better balance between FP and TP, avoiding overoptimization of TP at the expense of specificity and model generalization. We believe that increasing only TP while ignoring FP is not appropriate in drug screening. On the contrary, maintaining relatively high specificity ensures that the model focuses more on biologically significant binding sites. This prevents nonfunctional residues from being misclassified as calcium-binding sites. This approach helps concentrate efforts on high-quality targets during subsequent drug development stages, further improving development efficiency.

### The Performance Comparison with Different PCA Dimensions on the Testing Data Set

In the previous discussion, we noted that ProtTrans achieves a more stable trade-off between “enhancing sensitivity” and “maintaining high specificity.” In light of this, our subsequent dimensionality reduction experiments focused on the ProtT5-XL-UniRef50 model.

We performed PCA on the original 1024-dimensional ProtTrans vectors, reducing them to 64, 128, 256, 512, and retaining the initial 1024 dimensions. Our aim was to investigate whether it was possible to lower computational complexity, speed up training, or achieve better generalization. This was without losing too much critical information. According to Table 9 and Figure 5(c), while reducing dimensions causes minimal changes in TP, FP fluctuates considerably, and overall performance also varies significantly. Although using fewer dimensions requires fewer resources than the original 1024 dimensions, predictive accuracy (AUC) drops noticeably from 0.9718 to 0.9380.

**Table 9:** Performance Comparison with Different PCA Dimensions on the Testing Data Set

dimension	TP	FP	TN	FN	sensitivity	specificity	accuracy	MCC	AUC
original (1024)	26	80	3647	5	0.8387	0.9785	0.9774	0.4465	0.9718
64	26	264	3463	5	0.8387	0.9292	0.9284	0.2603	0.9380
128	25	149	3578	6	0.8065	0.9600	0.9588	0.3299	0.9333
256	26	314	3413	5	0.8387	0.9157	0.9151	0.2379	0.9471
512	25	196	3531	6	0.8065	0.9474	0.9462	0.2898	0.9458

Although dimensionality reduction can indeed reduce computational overhead and overfitting risk, in our study, balancing TP and specificity is pivotal for drug development. Sacrificing specificity to raise TP is particularly disadvantageous for drug discovery, as more false positives can lead to resource wastage and reduce overall predictive accuracy, introducing extra costs and unnecessary directions for subsequent screening and experiments.

Therefore, to achieve higher predictive accuracy and balance, the original 1024-dimensional ProtTrans still delivers better performance.

### The Performance Comparison with Merged Features on the Testing Data Set

In terms of model merging, considering that the 1280-dimensional ESM-2 model performs better than the 2560-dimensional version, we chose to use ESM-2-650M as the basis for comparison. We combined ProtT5-XL-UniRef50 with the other two embedded models, resulting in four combinations: (ProtT5-XL-UniRef50+ESM-2-650M), (ProtT5-XL-UniRef50+TAPE), (ESM-2-650M+TAPE), and (ProtT5-XL-UniRef50+ESM-2-650M+TAPE).

As shown in Table 10 and Figure 5-d, while these combinations slightly improve sensitivity in certain cases, they also significantly increase false positive rates. Although merging embeddings allows the model to capture more contextual information from sequences, it also introduces additional noise, resulting in reduced predictive accuracy. As mentioned in our discussion of ProtTrans advantages, an increase in TP often brings higher FP. While accurately predicting calcium-binding sites is crucial, an increase in FP indicates a higher likelihood of misclassification, leading to increased resource wastage and negatively impacting the model’s generalization ability.

**Table 10:** Performance Comparison with Merged Features on the Testing Data Set

feature sets	dimension	TP	FP	TN	FN	sensitivity	specificity	accuracy	MCC	AUC
ProtT5-XL-UniRef50	1024	26	80	3647	5	0.8387	0.9785	0.9774	0.4465	0.9718
ProtT5-XL-UniRef50+ESM-2-650M	2304	27	211	3516	4	0.8710	0.9434	0.9428	0.3024	0.9636
ProtT5-XL-UniRef50+TAPE	1792	27	322	3405	4	0.8710	0.9136	0.9133	0.2445	0.9523
ESM-2-650M+TAPE	2048	26	263	3464	5	0.8387	0.9294	0.9287	0.2608	0.9543
ProtT5-XL-UniRef50+ESM-2-650M+TAPE	3072	27	308	3419	4	0.8710	0.9174	0.9170	0.2502	0.9442

In this study, we emphasize the balance between TP and FP. The original ProtTrans embedding maintains the highest overall balance among all performance indicators, particularly in terms of AUC (0.9718) and MCC (0.4465). Therefore, ProtTrans alone emerges as our optimal choice.

### The Performance Comparison with Different Classifiers On The Testing Data Set

**Figure 6:**
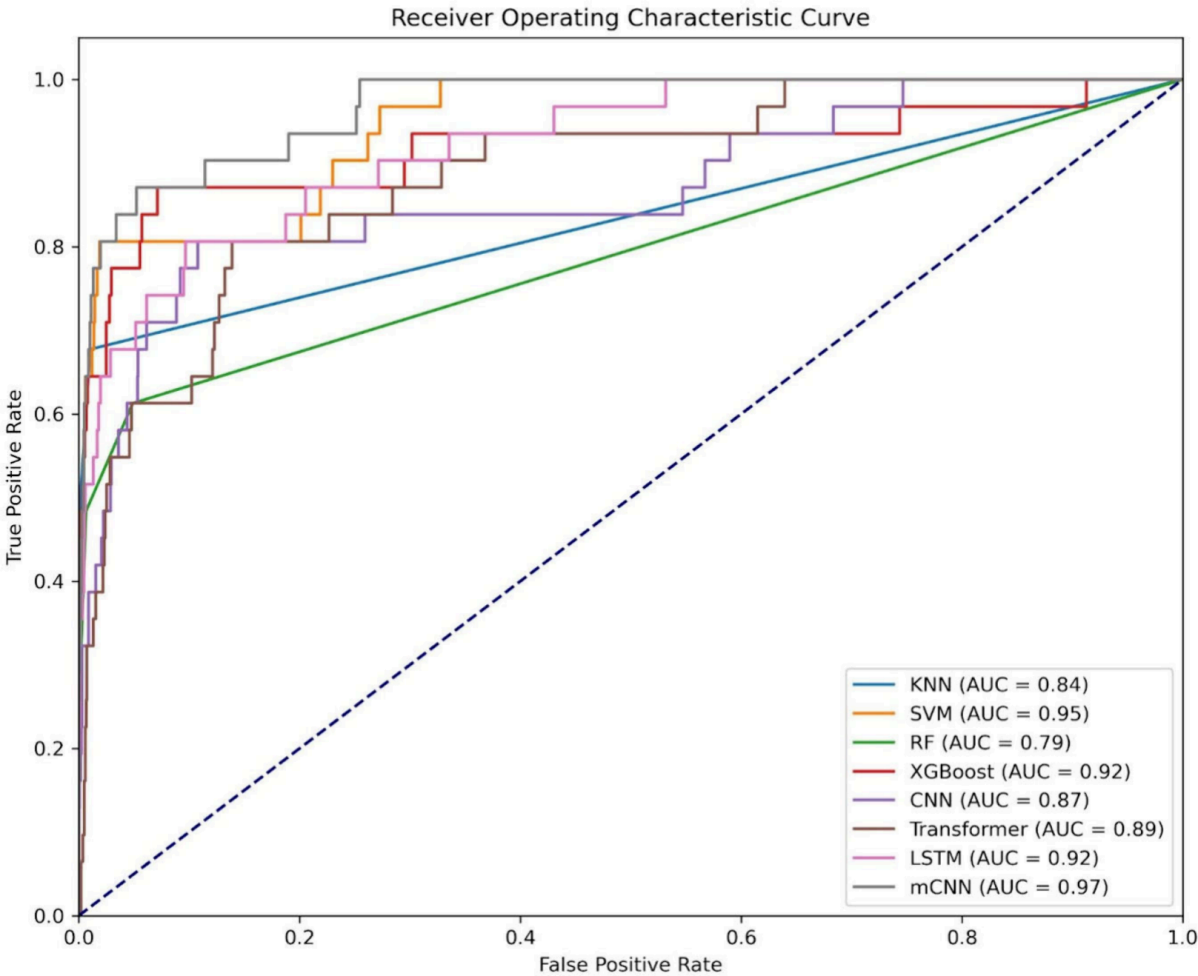
ROC curve for performance with different machine learning models.

Table 11 and Figure 6 compare mCNN’s performance with various deep learning and classification models. The TP (True Positive) values for KNN, SVM, RF, and XGBoost were 14, 11, 4, and 6, respectively, while their specificity values were 0.9987, 0.9984, 0.9995, and 0.9995. Although these models demonstrated excellent specificity (particularly RF and XGBoost, with specificity nearing 0.9995), their sensitivity was generally low, resulting in the inability to effectively detect real binding sites. RF and XGBoost achieved TP values of only 4 and 6, indicating their limited ability to recognize true binding sites. In drug target discovery, insufficient sensitivity may cause the model to miss many true positive cases. This may lead to overlooking critical binding sites and negatively impact drug development.

**Table 11:** Performance Comparison with Different Classifiers on the Testing Data Set

model	TP	FP	TN	FN	sensitivity	specificity	accuracy	MCC	AUC
KNN	14	5	3722	17	0.4516	0.9987	0.9941	0.5742	0.8358
SVM	11	6	3721	20	0.3548	0.9984	0.9931	0.4761	0.9474
RF	4	2	3725	27	0.1290	0.9995	0.9923	0.2911	0.7924
XGBoost	6	2	3725	25	0.1935	0.9995	0.9928	0.3788	0.9167
CNN	25	403	3324	6	0.8065	0.8919	0.8912	0.1988	0.8684
transformer	25	517	3210	6	0.8065	0.8613	0.8608	0.1719	0.8869
LSTM	25	360	3367	6	0.8065	0.9034	0.9026	0.2117	0.9227
mCNN	26	80	3647	5	0.8387	0.9785	0.9774	0.4465	0.9718

Compared to the four models mentioned above, CNN, Transformer, and LSTM significantly improved sensitivity and TP. However, this often came at the cost of higher FP. However, mCNN demonstrated significant advantages by achieving the highest TP (26) and substantially reducing FP (80) among all models. mCNN not only accurately identified calcium-binding sites but also effectively reduced the false positive rate for noncalcium-binding sites. An increase in TP indicates that the model can more precisely locate calcium-binding sites, while a reduction in FP minimizes the risk of misidentifying irrelevant sites as targets during drug screening and development, ultimately saving resources and improving drug development success rates.

In summary, compared to other models, mCNN validates features more effectively through its multiwindow design, capturing sequence features at different scales and avoiding information loss from single-window approaches. This feature extraction method enhances overall predictive accuracy, making the model more adaptable when dealing with complex and diverse protein sequences. It can identify more potential calcium-binding sites while minimizing false positives, thereby supporting the development of CCB drugs.

### The Performance Comparison with Previous Works

We compared its performance against four existing calcium-binding site prediction methods: DELIA,^30^ GraphBind,^24^ MIB2,^31^ and SCAMPER.^25^ DELIA uses a hybrid deep learning approach combining 1D sequence and 2D structural information, employing techniques like oversampling and undersampling to address imbalanced data sets. DELIA effectively integrates sequence and structural information and addresses data set imbalances; however, it struggles to capture global sequence patterns and may miss diverse binding sites due to its focus on local features. GraphBind leverages graph neural networks (GNNs), converting protein structures into graphs and using hierarchical GNNs to incorporate structural and physicochemical features. GraphBind excels at capturing structural motifs and physicochemical properties. However, its sensitivity to diverse binding sites is limited due to a pronounced bias toward specific structural motifs. MIB2 employs a fragment transformation method, comparing query protein regions to template structures from a metal ion-binding database. MIB2 effectively matches query regions to template structures but is constrained by the template database, potentially failing to identify novel or uncommon binding sites. SCAMPER utilizes a two-layer architecture, processing sequence data from the BioLip and UniProt databases and aiming to minimize cross-prediction errors between different interacting residue types. SCAMPER reduces cross-prediction errors and leverages large sequence databases; however, it may struggle with complex structural contexts and is highly dependent on input data quality.

CaBind_MCNN integrates embeddings from pretrained protein language models (pLMs) such as ProtTrans, ESM-2, and TAPE. These embeddings capture rich contextual information from protein sequences, providing comprehensive protein feature representations. This enhances the model’s ability to identify calcium-binding sites with high sensitivity and specificity. CaBind_MCNN employs a multiwindow convolutional neural network (mCNN) architecture, scanning protein sequences with variable-scale windows. This multiscale approach allows the model to capture both local and global sequence patterns. By integrating pLM embeddings, CaBind_MCNN leverages advanced feature representations and a robust model architecture, resulting in superior performance across various evaluation metrics.

As shown in Table 12, all models were evaluated based on a testing data set containing 31 calcium-binding residues and 3727 non binding residues, as well as sensitivity, specificity, accuracy, and Matthews Correlation Coefficient (MCC).

**Table 12:** Performance Comparison with Previous Methods

methods	years	sensitivity	specificity	accuracy	MCC
DELIA^30^	2020	0.3548	0.9995	0.9941	0.5458
Graphbind^24^	2021	0.0968	0.9987	0.9912	0.1538
MIB2^31^	2022	0.7097	0.8868	0.8853	0.1671
SCAMPER^25^	2023	0.2581	0.8887	0.0194	0.0425
CaBind_MCNN	2024	0.8387	0.9785	0.9774	0.4465

CaBind_MCNN achieved a sensitivity of 0.8387, significantly outperforming the other methods (DELIA: 0.3548, GraphBind: 0.0968, MIB2:0.7097, SCAMPER: 0.2581). Given the highly imbalanced nature of the data set, prioritizing sensitivity is crucial for effective calcium-binding site prediction. High sensitivity is essential for identifying the relatively rare true binding sites, paramount for downstream applications like CCB drug target identification. While CaBind_MCNN maintains high specificity (0.9785), its substantial sensitivity advantage underscores its effectiveness.

Despite exhibiting high specificity (0.9995), accuracy (0.9941), and MCC (0.5458), DELIA’s comparatively low sensitivity (0.3548) limits its practical utility. Its strategies for handling imbalanced data, while potentially beneficial for overall performance, may sacrifice the ability to capture a significant portion of true binding sites. GraphBind, despite similar high specificity (0.9987) and accuracy (0.9912), also suffers from extremely low sensitivity (0.0968). This suggests its graph-based approach may not effectively capture relevant binding features. MIB2, with a sensitivity of 0.7097, performs better than DELIA and GraphBind but remains inferior to CaBind_MCNN. Its template-based approach may struggle with proteins exhibiting novel structures or significant deviations from known templates. SCAMPER shows considerably lower performance across all metrics, including very low sensitivity (0.2581) and accuracy (0.0194), indicating limitations in its predictive capabilities.

Furthermore, CaBind_MCNN demonstrated a balanced approach, effectively identifying binding sites across diverse structural contexts without overly relying on specific structural features. In contrast, GraphBind showed a pronounced bias toward certain structural motifs, resulting in lower sensitivity. While DELIA and MIB2 exhibited more balanced performance, they still fell short of capturing the full spectrum of true binding sites compared to CaBind_MCNN. This comprehensive performance across varied structural scenarios highlights CaBind_MCNN’s robustness and adaptability to calcium-binding site prediction.

Our emphasis on sensitivity reflects the specific demands of calcium-binding site prediction for drug discovery. While CaBind_MCNN’s MCC (0.4465) is slightly lower than DELIA’s, the substantial gain in sensitivity is a worthwhile trade-off. Accurately identifying true binding sites, even at the cost of a marginal increase in false positives (and a slightly lower MCC), is crucial for effective drug target identification. This focus on sensitivity reinforces CaBind_MCNN’s practical value in accelerating the discovery of potential CCB drug targets.

### Enhanced Validation Using a ChEMBL Data Set of CCB Targets

To further validate our model and address potential concerns about the initial data set size, we curated a new data set from the ChEMBL database^32^ focusing on calcium channel blockers (CCBs)^33^ and their protein targets. Starting with a search for “Calcium Channel Blocker,″ we identified 315 related proteins. We then filtered this set to include only proteins with annotated binding sites, resulting in 50 proteins. We used CD-HIT with a 40% similarity threshold to process the data against the training set, preventing overfitting caused by overly similar data. This resulted in a data set containing 23 calcium ion channel protein sequences. The data set includes 23,817 residues, of which 177 are calcium-binding sites and 23,640 are nonbinding sites.

This ChEMBL-derived data set offers several advantages. Given that CCBs like Nitrendipine and Diltiazem primarily target L-type calcium channels, focusing on CCB-interacting proteins increases the pharmacological relevance of our validation. By accurately predicting calcium-binding sites within these proteins, we aimed to improve the model’s ability to identify potential drug targets and facilitate the development of more effective CCBs. For example, the data set includes proteins like the L-type calcium channel Q13936, enabling analysis of its binding interactions and contributing to a deeper understanding of the structural features essential for drug binding and channel inhibition.

**Figure 7:**
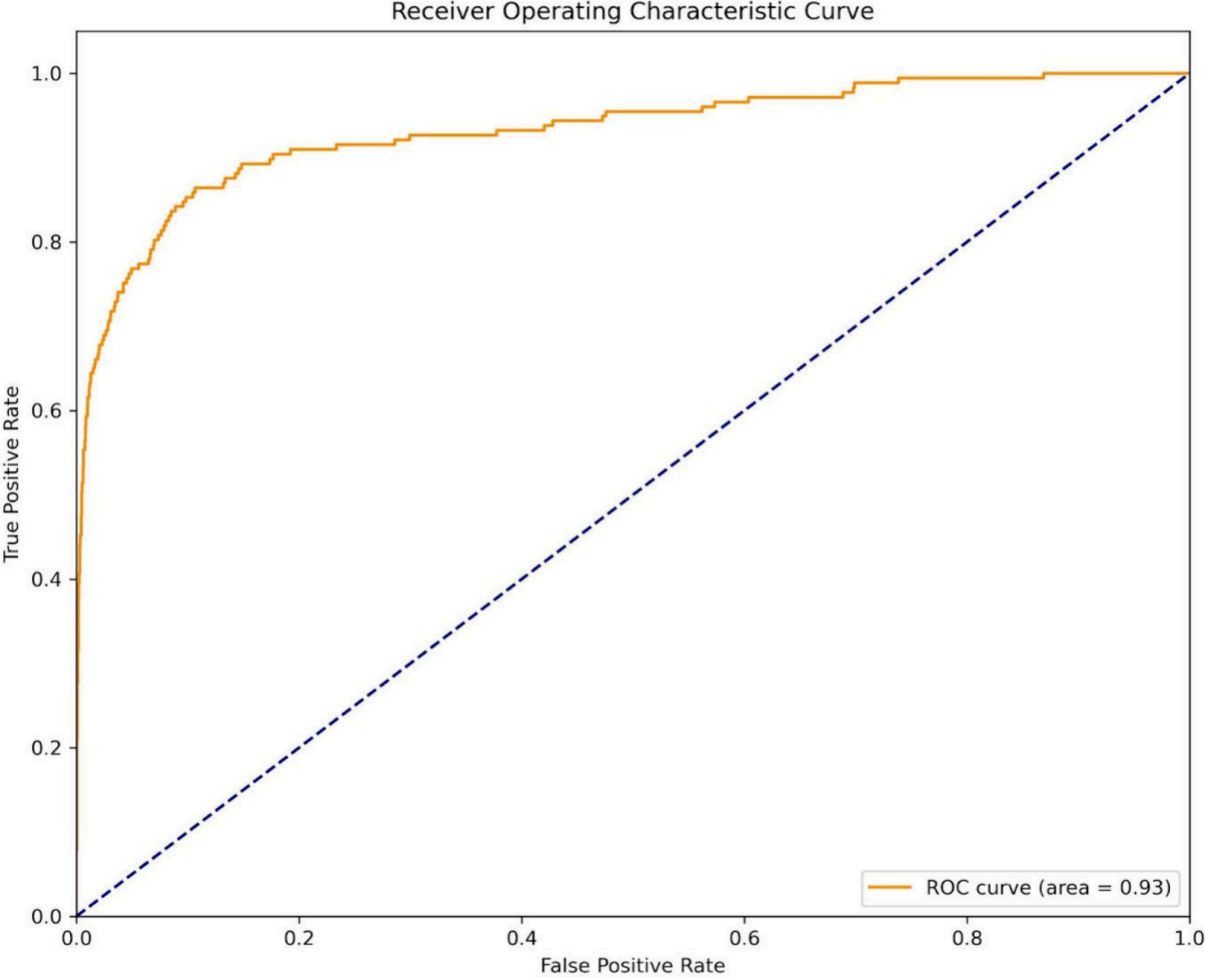
ROC curve for the performance of predicting calcium interacting residues from calcium channel with the ChEMBL data set.

As shown in Table 13 and Figure 7, our model achieved a sensitivity of 0.8644 on this ChEMBL data set, demonstrating its ability to accurately predict calcium-binding sites in pharmacologically relevant calcium ion channels. This high sensitivity underscores the model’s potential to accelerate CCB drug discovery. While the MCC is relatively low, our primary focus remains sensitivity because the interaction of CCBs with calcium-binding sites is paramount for drug target identification. This refined data set allows for a more accurate and contextually relevant assessment of our model’s ability to predict calcium-binding sites, leading to a deeper understanding of calcium ion-protein interactions and potentially facilitating the design of more targeted and effective CCB drugs.

**Table 13:** Enhanced Validation Using a ChEMBL Data Set of CCB Targets

data set	sensitivity	specificity	accuracy	MCC	AUC
independent test with ChEMBL data set	0.8644	0.8934	0.8932	0.2062	0.9319

## Conclusion

In this study, we present a new computational model called CaBind_MCNN, designed to identify calcium-binding sites within ion channels and transporter proteins. By combining protein language models (PLMs) with multiscale feature extraction methods, our model has demonstrated outstanding performance in predicting calcium-binding sites, achieving a high AUC of 0.9886. This exceptional predictive capability significantly surpasses traditional methods, highlighting the immense potential of our model in both basic research and practical drug discovery.

There are significant practical implications to our findings. Calcium ions play a crucial role in various physiological processes. Dysregulation of calcium homeostasis is closely associated with several serious health issues, including arrhythmias, hypertension, and other cardiovascular diseases. Ion channels and transporter proteins are key to maintaining intracellular calcium ion balance, and disruption in the function of these proteins can lead to severe pathological conditions. Our model’s ability to accurately predict calcium-binding sites in these proteins provides a powerful tool for understanding the molecular mechanisms underlying these diseases.

Moreover, the current limitations of existing calcium channel blockers (CCBs) in clinical practice further highlight the relevance of our work. While CCBs have been effective in treating various cardiovascular conditions, their use is often limited by side effects, such as hypotension, dizziness, and reduced cardiac contraction which can be particularly detrimental in patients with heart failure. Additionally, the interaction of CCBs with other medications can lead to adverse outcomes, such as excessive bradycardia and atrioventricular conduction block. These challenges underscore the need for improved, more selective, and safer CCBs.

The CaBind_MCNN model holds significant promise in addressing these challenges. By accurately identifying calcium-binding sites, our model can facilitate the discovery of novel drug targets, potentially leading to the development of innovative CCBs that are more effective and have fewer side effects. This could include the design of medications specific to certain calcium channel subtypes or the optimization of existing CCBs to enhance their therapeutic profile. The insights gained from our model could also contribute to a deeper understanding of the mechanisms of action of current medications, enabling the refinement of existing therapies to better meet patient needs.

**Key Points**
1. CaBind_MCNN identifies potential CCB targets: The model predicts Ca^2+^ binding sites in ion channels and transporter proteins, highlighting potential targets for novel CCB development.
2. Leverages advanced protein language models: CaBind_MCNN utilizes ProtTrans, a powerful PLM, to generate enriched sequence representations capturing crucial contextual information for accurate binding site prediction.
3. Employs multiscale feature extraction: The multiwindow scanning approach of CaBind_MCNN effectively captures local and global sequence features relevant to Ca^2+^ binding, improving prediction accuracy.
4. Outperforms existing methods: CaBind_MCNN achieves a high AUC of 0.9886, surpassing existing Ca^2+^ binding site prediction methods, particularly in sensitivity.
5. Facilitates drug discovery for calcium-related disorders: By identifying potential CCB targets, CaBind_MCNN can accelerate the development of novel therapies for diseases linked to Ca^2+^ dysregulation, such as arrhythmias and hypertension.

## Data Availability

In addition, the code and data for this work are available at: https://github.com/B1607/CaBind_MCNN
